# Oxalate of Cerium as a Remedy in Cough

**Published:** 1880-08

**Authors:** 


OXALATE OF CERIUM AS A REMEDY IN COUGH, Is attracting some attention, and from our experience we think it entitled to consideration as a remedy in chronic cough, as in bronchitis, phthisis, etc. It does not offend the stomach as most cough remedies are apt to do, and does not consequently interfere with other treatment necessary in a case requiring its use. It may be given in doses of from five to ten grains, three times a day. It usually requires two or three days for its effects to become manifest, and they pass away in about the same length of time, after the disuse of the remedy.

YELLOW FEVER.—In view of the vast importance of the subject, and the arrival of the season during which its advent is most to be apprehended, we would respectfully solicit contributions from gentlemen who have had *practical* experience with the disease. We desire to lay such information before our readers, as will lead to more rational and satisfactory ideas of its origin, and essential nature, than obtains with the inexperienced portion of the profession at the present time. While we hold opinions in regard to yellow fever, of a very decided character, which are known to many of the profession, we desire all the light and information that can be brought to bear upon it for the good of the profession generally, especially in the communities most exposed to its devastating visitations. Dr. Hargis, of Pensacola, Florida, was heard from in our last issue, and we trust other gentlemen will find time and inclination to enlighten our readers with their experience and views.

